# Insight in obsessive-compulsive disorder: conception, clinical characteristics, neuroimaging, and treatment

**DOI:** 10.1093/psyrad/kkad025

**Published:** 2023-11-08

**Authors:** Yueqi Huang, Yazhu Weng, Lan Lan, Cheng Zhu, Ting Shen, Wenxin Tang, Hsin-Yi Lai

**Affiliations:** Department of Psychiatry, Affiliated Mental Health Center and Hangzhou Seventh People's Hospital, Zhejiang University School of Medicine, Hangzhou 310007, China; Fourth Clinical School of Zhejiang Chinese Medical University, Hangzhou 310053, China; Department of Psychology and Behavior Science, Zhejiang University, Hangzhou 310058, China; Department of Psychiatry, Affiliated Mental Health Center and Hangzhou Seventh People's Hospital, Zhejiang University School of Medicine, Hangzhou 310007, China; Frontotemporal Degeneration Center, Department of Neurology, Perelman School of Medicine, University of Pennsylvania, Philadelphia 19104, PA, USA; Department of Psychiatry, Affiliated Mental Health Center and Hangzhou Seventh People's Hospital, Zhejiang University School of Medicine, Hangzhou 310007, China; Department of Psychiatry, Affiliated Mental Health Center and Hangzhou Seventh People's Hospital, Zhejiang University School of Medicine, Hangzhou 310007, China; Department of Neurology of the Second Affiliated Hospital, Interdisciplinary Institute of Neuroscience and Technology, Key Laboratory of Medical Neurobiology of Zhejiang Province, Zhejiang University School of Medicine, Hangzhou 310029, China; MOE Frontier Science Center for Brain Science and Brain-Machine Integration, State Key Laboratory of Brain-machine Intelligence, School of Brain Science and Brain Medicine, Zhejiang University, Hangzhou 311121, China; College of Biomedical Engineering and Instrument Science, Zhejiang University, Hangzhou 310027, China

**Keywords:** obsessive-compulsive disorder, insight, mental health, neuroimaging

## Abstract

Obsessive-compulsive disorder (OCD) is a chronic disabling disease with often unsatisfactory therapeutic outcomes. The fifth edition of the *Diagnostic and Statistical Manual of Mental Disorders* (DSM-5) has broadened the diagnostic criteria for OCD, acknowledging that some OCD patients may lack insight into their symptoms. Previous studies have demonstrated that insight can impact therapeutic efficacy and prognosis, underscoring its importance in the treatment of mental disorders, including OCD. In recent years, there has been a growing interest in understanding the influence of insight on mental disorders, leading to advancements in related research. However, to the best of our knowledge, there is dearth of comprehensive reviews on the topic of insight in OCD. In this review article, we aim to fill this gap by providing a concise overview of the concept of insight and its multifaceted role in clinical characteristics, neuroimaging mechanisms, and treatment for OCD.

Obsessive-compulsive disorder (OCD) is a chronic disabling disease for its limited responses to both medical treatment and psychotherapy. Epidemiologic studies have indicated a lifetime prevalence of OCD ranging from 2 to 3% (Stein *et al*., 2019), and the latest research reports a lifetime prevalence of 2.4% in mainland China (Huang *et al*., 2019). While OCD affects both genders, the proportion of men experiencing OCD during childhood is higher, whereas women tend to be more affected during adolescence and adulthood (Mathes *et al*., 2019). The onset of OCD commonly occurs around puberty (13–18 years) and early adulthood (Albert *et al*., 2015; Anholt *et al*., 2014). OCD is characterized by intrusive obsessions and/or compulsions. Obsessions entail recurring, uncontrollable thoughts, images, impulses, or urges often accompanied by anxiety. Compulsions are repetitive, ritualistic behaviors aimed at relieving or preventing anxiety. Clinically, OCD frequently co-occurs with other symptoms such as anxiety, phobia, and other negative feelings. Historically, OCD has long been categorized as an anxiety disorder due to the prevalence of anxiety symptoms among patients (Stein *et al*., ). However, anxiety is not a necessary condition for an OCD diagnosis. Consequently, the fifth edition of the *Diagnostic and Statistical Manual of Mental Disorders* (DSM-5) categorizes OCD within an independent category called “obsessive-compulsive and other related disorders” (Fig. 1) (Gnanavel and Robert, 2013).

**Figure 1:**
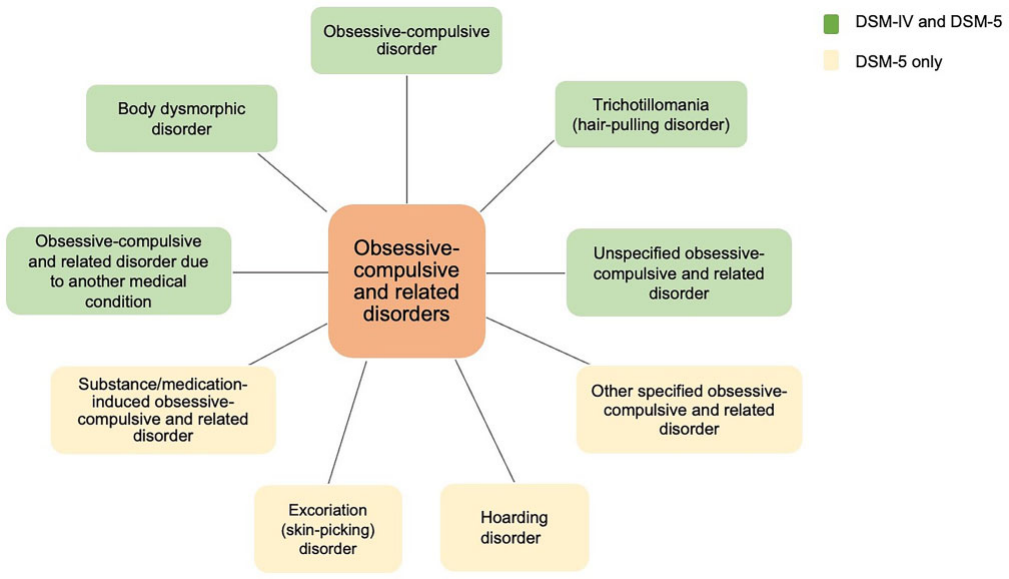
Obsessive-compulsive and related disorders. The obsessive-compulsive and related disorders chapter in the DSM-5 adds four items including substance/medication-induced obsessive-compulsive and related disorder, other specified obsessive-compulsive and related disorder, as well as hoarding disorder and excoriation (skin-picking) disorder.

The etiology of OCD is extremely complex, involving social, psychological, and biological factors. However, the fundamental pathogenesis of OCD remains unclear, complicating treatment. Prolonged OCD can significantly affect the daily lives and social functions of individuals (Thorsen *et al*., 2018). Current Canadian clinical practice guidelines recommend selective serotonin reuptake inhibitors (SSRIs) and cognitive behavioral therapy (CBT) as the first-line treatments (Katzman *et al*., 2014). Despite the primary reliance on medications in OCD treatment, specific drugs are lacking, and some available medications result in intolerable side effects. Consequently, 40–60% of OCD patients poorly respond to serotonin reuptake inhibitors (SRIs), and about two-thirds of those who switch to another SRI fail to achieve optimal therapeutic outcomes (Pallanti *et al*., 2002; Pallanti and Quercioli, 2006). Numerous studies have demonstrated that the role of CBT, a prominent psychotherapy technique, in OCD treatment. Some even argue that CBT may surpass medical treatment in efficacy (Ost *et al*., 2015), with response rates of 85% in both adults and children (Storch *et al*., 2009). However, widespread use of CBT is limited, especially in low- and middle-income countries, due to a shortage of trained psychotherapists for OCD. Moreover, the effectiveness of CBT can be hindered by patients’ lack of motivation, compliance, mental consciousness, cognitive abilities and insight, and family cooperation (Knopp *et al*., 2013). In addition, about 75% OCD patients have comorbid conditions such as depression, bipolar disorder, anxiety, eating disorder, substance abuse, Tourette syndrome, schizophrenia, and OCD-related disorders (Choi, 2009). These comorbidities not only interact with OCD but also significantly affect its treatment efficacy (He, 2017). While overall OCD treatment is effective, individual responses vary greatly, and residual symptoms, easy recurrence, and prolonged courses of the illness can negative affect patients’ quality of life and social functioning (Yazici and Percinel, 2015), as well as increase the economic burden of treatment (Strouphauer *et al*., 2023).

According to the DSM-5 diagnostic criteria, some OCD patients may lack insight into their condition, broadening the scope of OCD diagnosis. DSM-5 clarifies that the absence of insight or the presence of delusional beliefs should be categorized as OCD or a related disorder rather than a psychotic disorder (Gnanavel and Robert, 2013). Previous study has indicated that OCD patients with poor insight often experience more severe symptoms, exhibit poorer responses to medical treatment, face greater impairment in their social functioning leading to increased unemployment (Macy *et al*., 2013), and impose additional economic and social burdens on their families and society (Hollander *et al*., 2016). Approximately 13–36% of OCD patients lack insight (Manarte *et al*., 2021a), exacerbating the disease severity and complicating treatment and the social burden. Consequently, enhancing the insight of OCD patients is crucial to address the limitations of OCD therapy (Inanc and Altintas, 2018).

In summary, while studies have underscored the importance of insight in OCD patients, there is currently a lack of comprehensive integration of these literatures. In this article, we aim to bridge this gap by providing a comprehensive review encompassing the clinical characteristics of OCD insight, neuroimaging mechanisms, and progress in the treatment of OCD (Fig. 2).

**Figure 2:**
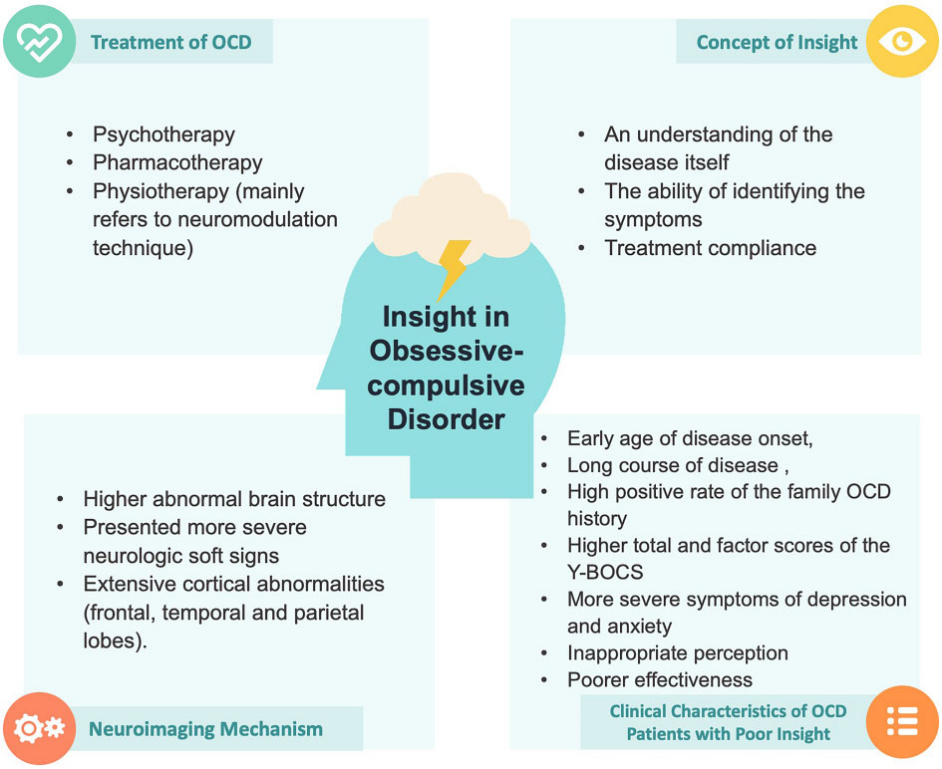
Overall discovery of the insight effect for OCD.

## Concept of insight

The define of insight has long been controversial. One widely accepted perspective in both clinical and scientific field involves patients becoming aware of their mental health issues, recognizing that these experiences are abnormal and require professional treatment (David, 2020). This concept comprises three core components: (i) understanding the nature of the disease itself; (ii) the ability to identify the associated symptoms; and (iii) treatment adherence, as proposed by David. Importantly, this concept applies to many psychiatric disorders (David, 2020).

However, not all patients agree with this viewpoint, some argue what is termed “insight” a merely amounts to agreeing with the doctor's opinion, which they perceive as cognitively unjust, discriminatory, and exclusionary toward knowledgeable individuals and their autonomy, infringing on their right to self-determination and self-expression (Slade and Sweeney, 2020). Consequently, they advocate for the use of trauma-informed methods and a mental health system that responds to individuals’ extreme experiences by listening and exploration rather than negating the reality of their experiences. Conversely, some individuals at risk of mental health issues generally accept definition of insight and are willing to receive “psychiatric emergency services” where temporary decisions are made by a psychiatrist acting as an agent. Future research could explore the possibility of involving both professionals and individuals with specific mental disorders in clinical interpretation of insight. This approach aims to achieve a consensus through objective evaluation, thereby providing better services for patients.

Research on the relationship between insight and mental illnesses began about 30 years ago (David, 2020). However, during that time, a *Lancet* editor commented on this research as “academically meaningful but clinically meaningless.” Currently, as more research has been conducted, several highly reliable and effective evaluation methods have emerged (Beck *et al*., 2004; Kaplan *et al*., 2006). For example, a conceptual analysis review suggests that insight is best regarded as a mental state, independent of any specific disease, rather than as a symptomatic dimension of the disorder (Markova *et al*., 2009). Furthermore, a set of reliable tools were used to analyze the correlation of insight with compliance and prognosis (Lincoln *et al*., 2007; Noordraven *et al*., 2016). In general, a good insight in patients has a more significant impact on prognosis than treatment compliance (Lincoln *et al*., 2007). In recent years, with the advancement of the field of “metacognition,” Beck *et al*. (2004) introduced a novel concept known as cognitive insight. Cognitive insight refers to a cognitive pattern where individuals challenge their own thoughts, beliefs, and behaviors. A long-term follow-up study involving patients with first-onset mental illnesses found that those with better insight experienced fewer symptoms (O'Connor *et al*., 2017). This suggests that recognizing and enhancing insight are crucial aspects of mental health treatment.

## Clinical characteristics of OCD patients with poor insight

Insight with regards to OCD typically refers to the accuracy of beliefs or rationality concerning compulsions influenced by obsessions (Leckman *et al*., 2010). It primarily manifests as individuals being aware that their obsessions and compulsions are symptoms of OCD. For OCD patients with poor insight, they tend to believe that their OCD beliefs are likely or entirely true. Approximately 13–36% of OCD patients fall into this category (Jacob *et al*., 2014). Nearly two decades ago, in an effort to alert clinicians to the range of insights observed in OCD patients and to explore the relationship between insight and other aspects of psychopathology and treatment, it was suggested that a “poor insight” subtype should be included in the DSM, fourth edition (DSM-IV) field trial (Foa *et al*., 1995). It was not until a decade ago that the DSM-5 officially recognized a spectrum of insight in OCD, including patients with poor insight (Gnanavel and Robert, 2013). Insight has been recognized as an important clinical feature of OCD (Nissen and Parner, 2018), and may serve as predictive factor for successful treatment (Garcia *et al*., 2010). Studies have revealed specific clinical characteristics associated with OCD patients with poor insight, including an early onset of disease (Fontenelle *et al*., 2013), a prolonged course (Fontenelle *et al*., 2010), and a high rate of family history of OCD (Catapano *et al*., 2010). Insight has been found to be positively associated with age, and younger patients tended to have lower levels of insight (Lewin *et al*., 2010). The development of insight generally emerges along with the emergence of abstract thinking and formal operations during growth (Zetlin and Turner, 1985). The prevalence of childhood OCD is ~1–4% (Millet *et al*., 2004; Zohar, 1999), which may be linked to multi-domain impairments, including issue related to insight. This suggests that addressing poor insight, especially in younger children, is crucial for early recognition and treatment.

Furthermore, OCD patients with poor insight often exhibit a high level of the contamination/cleaning dimension (Cherian *et al*., 2012; de Avila *et al*., 2019), symmetry/ordering/counting dimension (Hazari *et al*., 2016; Jakubovski *et al*., 2011), and hoarding dimension (de Avila *et al*., 2019; Fontenelle *et al*., 2013). These associations suggest that insight levels may aid in distinguishing OCD subtypes (de Avila *et al*., 2019). However, there is no unified conclusion on whether insight has a relationship with different biological and predictive validities across different dimensions of OCD (Markova *et al*., 2009). For the other side, studies have indicated specific correlations between insight and the severity of symptoms with the same dimension (de Avila *et al*., 2019). For example, OCD patients with poor insight trend to have more severe symptoms, as reflected by higher and factorial scores on the Yale–Brown Obsessive-Compulsive Scale (Y-BOCS) (de Avila *et al*., 2019). Even after comprehensive treatment, patients with poor insight trend to have a lower reduction in their Y-BOCS score and relatively poor therapeutic outcomes (Raffin *et al*., 2009; Selles *et al*., 2020). This lack of insight often results in OCD patients failing to recognize the pathological nature of their obsessions and compulsions, leading them to invest less effort in resisting their symptoms, ultimately resulting in more severe symptoms and impairments.

In clinical practice, neuroleptic medications are frequently used for patients with poor insight (de Avila *et al*., 2019) suggesting that these patients may have dysfunction in dopaminergic neural circuits. Combining an atypical antipsychotic with SSRIs may exert a synergistic effect by enhancing serotonergic activity or combining their respective mechanisms of action to strengthen the dopaminergic neural circuits involved. In addition, patients with poor insight may exhibit more severe symptoms, sometimes even reaching a paranoid degree, which leads psychiatrists to consider using neuroleptics (Hagen *et al*., 2017; Tonna *et al*., 2016). However, even with the use of second-generation neuroleptics such as Risperidone and Aripiprazole to potentiate treatment, the effective rate for refractory OCD remains at only ~40–55% (Albert *et al*., 2016). Studies have also shown that OCD patients with poor insight often have low levels of empathetic concern and a reduced ability to recognize emotional states, especially negative emotions (Manarte *et al*., 2021a). This may explain why OCD patients with poor insight experience more severe symptoms of depression and anxiety (Altintas and Taskintuna, 2015), a higher propensity for suicide tendency, and comorbidities of major depressive disorder (Fontenelle *et al*., 2013), dysthymia (Raffin *et al*., 2009), bipolar disorder, and post-traumatic stress disorder (de Avila *et al*., 2019).

Furthermore, OCD patients with poor insight often exhibit inappropriate perception, particularly in visual and tactile perceptions. This often indicates that a poor therapeutic outcome may be linked to specific neurobiological factors and poor insight (Moritz *et al*., 2018; Shavitt *et al*., 2014). Studies have shown that the severity of perception abnormalities is positively correlated with the overall severity of OCD symptoms (Moritz *et al*., 2018). Inappropriate perception plays a significant role in inducing compulsions in ~65% OCD patients (Ferrao *et al*., 2012). Abnormal perception can increase the subjective reality of obsession (Jakubovski *et al*., 2011), thus exacerbating the difficulty in changing or resisting these symptoms (Moritz *et al*., 2018) (Table 1).

**Table 1:** Characteristics of OCD patients with poor insight.

	OCD with poor insight
Clinical characteristics	1. early age of disease onset
	2. long course of disease
	3. high positive rate of the family OCD history
	4. higher total and factor scores of the Y-BOCS
	5. more severe symptoms of depression and anxiety
	6. inappropriate perception
	7. poorer effectiveness
Dimension characteristics	1. high contamination/cleaning dimension
	2. high symmetry/ordering/counting dimension
	3. high hoarding dimension
Comorbidity characteristics	1. major depressive disorder
	2. dysthymia
	3. bipolar disorder
	4. post-traumatic stress disorder

From the neuropsychological aspect, OCD patients with poor insight often exhibit poorer verbal memory and executive function (Kitis *et al*., 2007; Tumkaya *et al*., 2009). While there is debate regarding verbal memory impairment, some researchers argue that OCD patients have impaired “organizational strategy” rather than verbal memory (Chamberlain *et al*., 2005), suggesting that the cognitive deficits in OCD patients with poor insight are related to more complex executive function (Manarte *et al*., 2021a). Executive function includes various cognitive processes, including working memory, cognitive flexibility, behavioral cognitive control, attention, self-control, and self-regulation of actions. These executive functions rely on a neural network involving the prefrontal cortex, striatum, and cerebellum, with the prefrontal cortex being the primary structure (McDougle *et al*., 2021; Tian *et al*., 2020; Wilson *et al*., 2021). In the following section, we further discuss neuroimaging changes in OCD patients with poor insight, aiming to provide valuable pathways for the treatment of the disorder.

## Neuroimaging changes related to OCD insight

Recent advancements in brain imaging technologies, particularly magnetic resonance imaging (MRI), have facilitated numerous neuroimaging studies focusing on brain abnormalities in psychosis with poor insight, especially OCD. These studies have revealed structural abnormalities, with most identifying irregularities in frontal, temporal, and parietal regions (Cooke *et al*., 2008; Buchy *et al*., 2017; Orfei *et al*., 2017). Functional neuroimaging research has also demonstrated that insight is associated with functional abnormalities in both medial and lateral frontal, temporal, and parietal regions (Raij *et al*., 2012; van der Meer *et al*., 2013; de Vos *et al*., 2015). Moreover, poorer insight has been correlated with reduced white matter volume across the brain and diminished gray matter volume in the frontal gyri (Pijnenborg *et al*., 2020).

Many studies have reported brain functional and structural changes in OCD patients (Bourne *et al*., 2012; Diwadkar *et al*., 2015; Posner *et al*., 2014). Our recent study has identified a significant relationship between brain function and the severity of obsessions, as well as between brain structure and the severity of compulsions in individuals with OCD. (Tang *et al*., 2023). However, the neurology of OCD insight remains less explored. Early studies indicated that OCD patients with poor insight exhibited greater structural brain abnormalities (Aigner *et al*., 2005) and more pronounced neurologic soft signs, suggesting more extensive neurodevelopmental abnormalities (Karadag *et al*., 2011). However, these studies identified only specific brain regions, including the basal ganglion region, parietal lobe (Aigner *et al*., 2005), and the temporal lobe (Fan *et al*., 2017a). Recent research had extended the scope to include the cortical structure, such as cortical thickness and local gyrification index, as well as brain networks in insight investigations (Liu *et al*., 2019). Notably, reduced cortical thickness in the dorsal medial prefrontal cortex, left anterior cingulate cortex (ACC), and right lateral parietal cortex was observed in OCD patients with poor insight compared to those with good insight (Liu *et al*., 2019). Reduced cortical thickness, representing a reduction in neurons within cortical columns, may reflect insight-related changes (Karadag *et al*., 2011). OCD patients tend to have hyperactive error-monitoring functions, leading to slower decision-making and delayed response to errors, known as “post-error slowing.” The medial prefrontal cortex, a key player in the error monitoring in OCD patients (Modirrousta *et al*., 2015), further supports the involvement of frontal lobe dysfunction in executive function in OCD patients with poor insight. Additionally, the ACC and orbitofrontal cortex (OFC) in the frontal lobe play roles in executive function in OCD patients (Manarte *et al*., 2021b). A study found a negative correlation between the insight score on the Y-BOCS and local gyrification index of the left OFC and left ACC (Venkatasubramanian *et al*., 2012). The ACC, a subcortical structure in the frontal lobe, is probably a high-level regulatory region for emotional information processing and behavioral planning and execution (Bush *et al*., 2000; Palomero-Gallagher *et al*., 2008), with the dorsal ACC primarily involved in the cognitive function of error monitoring (Bush *et al*., 2000).

Self-enhanced behavioral monitoring is considered an endophenotype of OCD. A 12-year follow-up study revealed that children with high levels of observational behavioral monitoring were twice as likely to develop OCD compared to a control group (Gilbert *et al*., 2018). This heightened behavioral observation was associated with a relatively smaller ACC volume on the right dorsal side (Gilbert *et al*., 2018), suggesting that the ACC plays a crucial role in modulating behavioral flexibility in response to changing environments (D'Cruz *et al*., 2011). This finding provides further insight into abnormalities observed in the ACC and LGL in OCD patients with poor insight. As part of the frontal lobe, the OFC contributes to executive function and is vital for human recognition, enhancing the learning process, and processing emotions. Overactivation of the OFC can lead to the emergence of repetitive thoughts (obsessions). OCD patients employ compulsions to alleviate anxiety and fear arising from the obsessions, with this repetitive behavior associated with the corpus striatum (Saxena and Rauch, 2000). Abnormal insight in schizophrenia and other psychiatric disorders is linked to defects in the medial OFC (Shad *et al*., 2006). In summary, the frontal cortex, likely through its involvement in executive function, plays a significant role in OCD insight. The parietal lobe, particularly lesions in the right hemisphere, has been associated with anosognosia (i.e. lack of awareness of neurological deficit symptoms) (Bisiach *et al*., 1986). Research conducted on schizophrenic patients with poor insight has reported that there is a decrease in the density of oligodendrocytes in the inferior parietal leaflet and reductions in the volume or thickness of the prefrontal and parietal lobes, which is associated with decreased self-reflective ability (i.e. cognitive insight). These findings suggest that alterations in brain regions involved in self-reflection and metacognition, such as the medial frontal and inferior parietal lobes, may play a role in the pathophysiology of poor insight in disorders such as schizophrenia. This insight could potentially be applicable to understanding obsessive-compulsive internality as well, given the shared features of insight-related abnormalities across these disorders (Buchy *et al*., 2016; Liu *et al*., 2019; Orfei *et al*., 2013).

Considering the fluctuating nature of insight (Abramowitz and Jacoby, 2015), functional MRI might hold more promise than structural MRI in revealing this process. Compared with OCD patients with good insight, those with poor insight exhibited dea creased amplitude of low-frequency fluctuation in the middle temporal gyrus and superior temporal gyrus (Fan *et al*., 2017b). The temporal lobe primarily participates in language processing, social perception, and semantic memory, and it is especially sensitive to information related to social relevance or significance (Hoekert *et al*., 2008; Kumari *et al*., 2010). Difficulties in transforming experiences into memories may hinder the assimilation of corrective information, contributing to the persistence of poor insight. A study on brain networks found that the functional connectivity between the anterior insula and right medial OFC was reduced in OCD patients with poor insight, and this reduction was negatively correlated with the level of insight (Fan *et al*., 2017a). Abnormal connectivity between the right anterior insula and right medial OFC mediated the poor insight in OCD, suggesting that impaired ability to encode and integrate self-assessment information (Fan *et al*., 2017a). In addition, research based on a small world model demonstrated a significant reduction in the length of theta wave paths in OCD patients with poor insight (Lei *et al*., 2017). This reduction may disrupt the balance of the small-world model network, leading to dissatisfaction with brain network topology and affecting the functional integration of theta waves in the brain network (Lei *et al*., 2017). The theta wave is known to play a role in working memory (Lisman, 2010). As a result, OCD patients with poor insight often exhibit more pronounced defects in working memory (Kashyap *et al*., 2012). Consequently, patients may persist in maintaining irrational beliefs, including the belief that their OCD symptoms are rational and appropriate (Kashyap *et al*., 2012). For example, patients with poor insight may persist in believing that their OCD symptoms are rational and appropriate.

In summary, OCD patients with poor insight appear to exhibit more extensive cortical abnormalities, involving the frontal, temporal, and parietal lobes. Previous studies on insight in schizophrenic patients have also shown that abnormal changes in the frontal, parietal, and temporal lobes are associated with poor insight (Buchy *et al*., 2017). These illnesses, including anosognosia, share structural similarities related to insight-related abnormalities, suggesting a common anatomical basis for poor insight in these conditions.

## Relationship between insight and treatment

**Figure 3:**
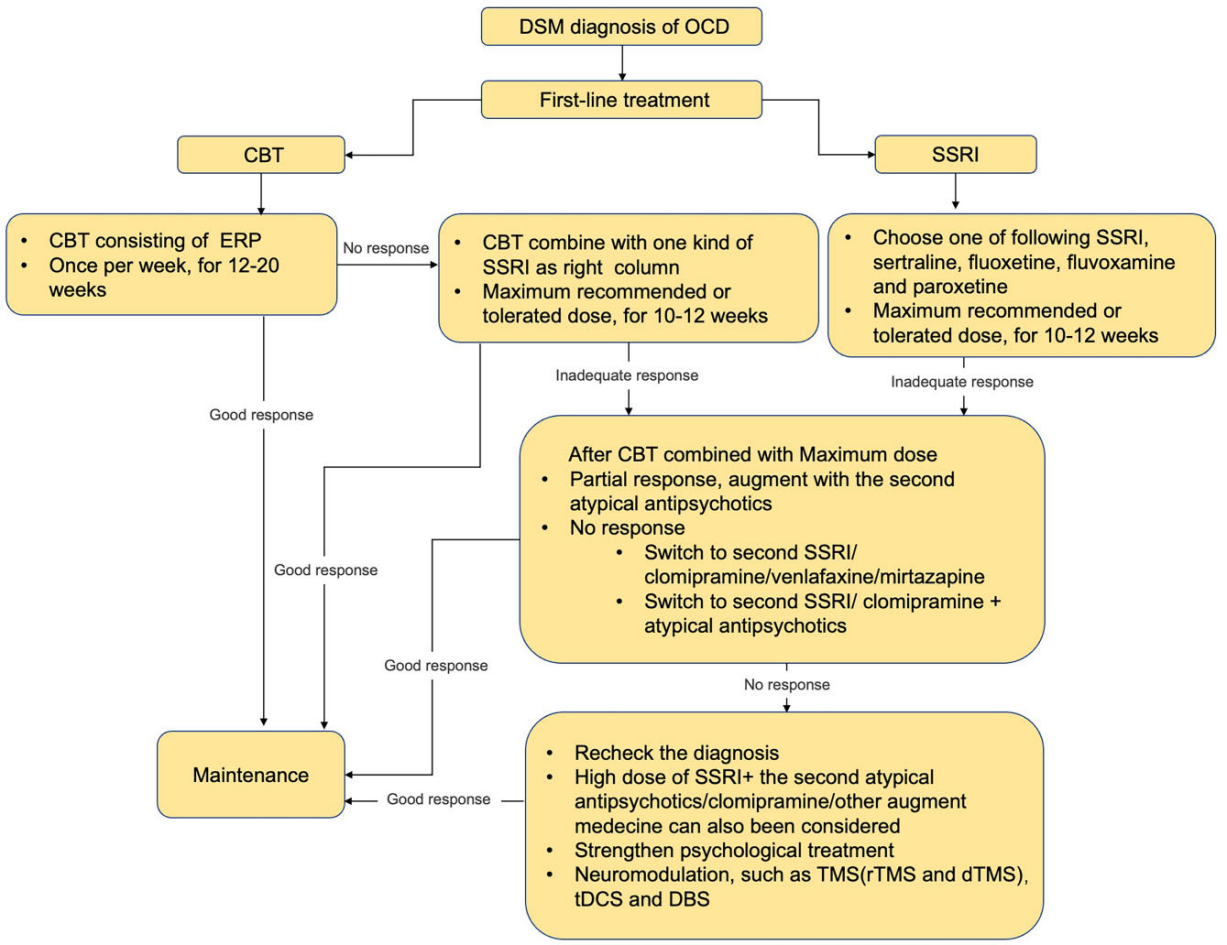
OCD treatment algorithm. tDCS, transcranial direct current stimulation.

Treatment of OCD encompasses psychotherapy, pharmacotherapy, and physiotherapy (mainly referring to a neuromodulation technique). Figure 3 shows an OCD treatment algorithm. Conventional treatments, such as psychotherapy and pharmacotherapy, have demonstrated effectiveness in enhancing the insight of OCD patients to a certain extent. CBT is a first-line treatment for OCD, and many studies have shown its efficacy in improving insight (Matsunaga *et al*., 2002; Selles *et al*., 2020). Contrary to generally accepted views on adult OCD, CBT treatment has shown no impact on baseline insight in pediatric OCD patients. This suggests that CBT is equally effective regardless of whether or not young patients recognize the excessive, impractical, or obsessive nature of their beliefs (Selles *et al*., 2020). Additionally, mindfulness-based cognitive therapy (MBCT) has proven beneficial for enhancing insight in OCD patients (Landmann *et al*., 2019). Mindfulness and acceptance-based strategies have been found to reduce pain associated with intrusive thoughts (Marcks and Woods, 2005; Najmi *et al*., 2009), increase a patient's willingness to experience intrusive thoughts (Marcks and Woods, 2007), and alleviate post-exposure anxiety and impulsive behaviors (Catapano *et al*., 2001). With respect to medical treatment, research indicates that poor insight can predict the therapeutic efficacy of SSRIs for OCD. However, while OCD symptoms and insight do improve some extent after treatment, the effect remains limited (Alonso *et al*., 2008; Catapano *et al*., 2001). Consequently, clinicians often turn to neuroleptics in the treatment of OCD patients with poor insight (de Avila *et al*., 2019). Second-generation neuroleptics such as Risperidone and Aripiprazole can potentiate the therapeutic effect in improving the insight in some patients, although their effectiveness in treating refractory OCD patients ranges from 40 to 55% (Albert *et al*., 2016). Moreover, structural abnormalities (Zhong *et al*., 2019) and altered functional connectivity (Viol *et al*., 2020; Yang *et al*., 2015) in related brain areas partially restored in OCD patients after treatment. This suggests that OCD treatment not only ameliorates symptoms but also affects the neurophysiology of relevant brain areas.

In recent years, various non-invasive neuromodulation technologies, such as transcranial magnetic stimulation (TMS), including repetitive TMS (rTMS) and deep TMS (dTMS), as well as transcranial direct current stimulation (Saba *et al*., 2015), have gained extensive use in treating a series of mental and neurological disease by regulating neural activity and plasticity (Matsumoto *et al*., 2010; van den Heuvel *et al*., 2009). Neuromodulation fundamentally aims to restore abnormal insight to the normal level by modulating abnormal brain function in OCD (Bais *et al*., 2014; Del *et al*., 2011). Studies have shown that rTMS can contribute to improving a patient's insight (Dlabac-de *et al*., 2015; Tateishi *et al*., 2019). According to the 2019 edition of the Harvard South Shore Program for OCD treatment, if the second-generation neuroleptics and other synergistic drugs are ineffective, highly safe and non-invasive neuromodulation techniques such as TMS can be considered in the next step, potentially elevating the priority of treatment to node 5 (Beaulieu *et al*., 2019). Compared with medical treatment, comprehensive treatment involving the combination of medical treatment, CBT, and rTMS targeting the left frontal lobe has demonstrated the ability to enhance insight in OCD patients, especially those with low baseline scores on the Brown Assessment of Beliefs Scale (Huang *et al*., 2022). In addition, transcranial direct current stimulation targeting the bilateral supplementary motor area has shown potential in improving OCD insight, with its effects not correlated with reductions in Y-BOCS scores (Harika-Germaneau *et al*., 2020). To target deeper subcortical structures and larger brain volumes, dTMS has been purposed to stimulate the medial prefrontal cortex and ACC: core regions that are associated with insight in OCD. While there is no direct evidence showing that dTMS improves OCD insight, the study has indicated that dTMS applied to the medial prefrontal cortex-ACC significantly improves Y-BOCS scores in OCD patients who failed to respond to medical treatment (Carmi *et al*., 2018). In addition, research suggests that high-frequency (20 Hz) dTMS is more effective in treating OCD symptoms compared to low-frequency dTMS (1 Hz). Deep brain stimulation (DBS) is an invasive physiotherapy for psychosis (Georgiev *et al*., 2021). A meta-analysis has demonstrated that DBS can significantly alleviate compulsion, depression, and anxiety symptoms in OCD patients who do not respond to medical treatment (Cruz *et al*., 2022). This improvement may be linked to the fact that cognitive dysfunction is a common adverse reaction to DBS. At present, there is limited research on the correlation between stimulation technologies and insight, which could be an interesting field for future research.

## Summary and perspectives

OCD is a chronic illness that can significantly affect a patient's social functioning and cause marked distress. Poor insight in patients is a crucial factor complicating the treatment of OCD. In clinical practice, insight serves as a vital metric for assessing the progress of mental illness treatment and plays a pivotal role in consolidating therapeutic effects and preventing recurrence. Although the conventional clinical definition of insight has been accepted by the public, some patients may perceive it simply as agreeing with their doctor's opinion. However, patients often bring their own biases rooted in their experience and knowledge, necessitating a fresh perspective in the concept of insight. From this perspective, insight is not a matter of presence or absence, or even a continuous spectrum. On the contrary, it represents a process of constructing personal meaning, which is limited over time by culture factors. A profound understanding of this concept empowers healthcare providers to better address a patient's extreme experience through active listening and exploration, a crucial aspect of effective disease treatment. Insight holds great importance in promoting treatment compliance. In the future, adopting a bottom-up approach (i.e. from life experience to theory) or engaging mental health professionals and patients collaboratively in narratives related to specific mental illness could prove advantageous. Starting with empathetic listening and understanding of patients’ experiences can substantially enhance treatment compliance.

Most studies concur that OCD patients with poor insight tend to exhibit early onset, a protracted disease course, severe symptoms, a family history of OCD, and a high prevalence of comorbidities. Present research suggests that insight may be linked to the contamination/cleaning, symmetry/ordering/counting, and hoarding dimensions. However, consensus remains elusive on dimensions related to poor insight. It is imperative to expand sample sizes and comprehensively assess drug-naive OCD patients at the onset. OCD patients with poor insight often coincide with distorted perceptions, highlighting that subjective reality of obsession and diminishing resistance to abnormal obsessions and compulsions. Such patients frequently exhibit cognitive biases, potentially reaching paranoid ideation, which explains the common use of neuroleptics in clinical practice.

In the diagnosis of mental illnesses such as OCD, it is crucial to rule out physical diseases with medical imaging techniques such as MRI. Additionally, patients often voluntarily undergo these procedures to understand the root causes of their mental health concerns. Therefore, patients voluntarily participating in the MRI examinations promote the blossoming of neuroimaging research. Neuroimaging studies have consistently implicated various brain regions, primarily the frontal, temporal, and parietal lobes, in the context of insight in OCD. These findings align with the involvement of key brain regions, including medial prefrontal cortex, ACC, and OFC, belonging to the critical cortico-striato-thalamo-cortical circuit: a fundamental model for understanding OCD pathophysiology. Many studies have reported fine structural changes in the cortico-striato-thalamo-cortical circuit, including alterations in gray matter volume (Rotge *et al*., 2010) and functional abnormalities (Posner *et al*., 2014; Zhang *et al*., 2017). These brain regions play pivotal roles in memory, emotional regulation, and cognitive processes. Their abnormalities often underlie the genesis of obsessions and the repetitive nature of symptoms (van den Heuvel *et al*., 2009; Nakao *et al*., 2014). Psychotherapy, pharmacotherapy, and physiotherapy have shown the potential to improve the insight of OCD patients (Huang *et al*., 2022; Landmann *et al*., 2019; Tateishi *et al*., 2019; Selles *et al*., 2020). Comprehensive treatment, combining medical treatment with CBT and rTMS targeting the left frontal lobe, has proven effective in enhancing OCD insight (Huang *et al*., 2022). Importantly, treatment outcomes are notably superior in patients with improved insight compared to those with poor insight (Huang *et al*., 2022). Therefore, enhancing insight in OCD patients can not only boost treatment efficacy but also enhance social functioning, alleviating the burden on families and society at large. With the advancements in physiotherapy and imaging technology, future research should intensify efforts to identify brain regions and circuits associated with insight, facilitating precise neuromodulation treatments. Some scholars have proposed that, as OCD patients’ stimulation targets are variable, selecting physiotherapy based on the specific symptom profile can activate frontal cortex regions more effectively (Barcia *et al*., 2019; Janssen *et al*., 2022). Future endeavors may involve larger-sample cohort studies, allowing for precise, individualized physiotherapy plans tailored to demographic data, clinical symptoms, and MRI findings. In recent years, governments worldwide and scientists have invested in R&D inbrain–computer interface,s artificial intelligence, and other cutting-edge technologies. These advancements hold the promise of creating more intelligent physical therapies. offering renewed hope for patients with OCD and other refractory mental illnesses.
